# Tetra­methyl anthracene-2,3,6,7-tetra­carboxyl­ate–tetra­methyl 9,10-dihydro-9,10-dioxoanthracene-2,3,6,7-tetra­carboxyl­ate (1/1)^1^

**DOI:** 10.1107/S1600536812032424

**Published:** 2012-07-25

**Authors:** Brenton L. Drake, J. Larry Morris, Mark L. McLaughlin, Frank R. Fronczek, Steven F. Watkins

**Affiliations:** a Department of Chemistry, Louisiana State University, Baton Rouge, LA 70803-1804, USA; b GEO Specialty Chemicals, WTC Process Technology Laboratory, Louisiana Business and Technology Center, 8000 GSRI Avenue, Building 3100, Baton Rouge, LA 70820, USA; c Department of Chemistry, 4202 E. Fowler Avenue, CHE 205, University of South Florida, Tampa, Florida 33620, USA

## Abstract

In the title co-crystal, C_22_H_16_O_10_·C_22_H_18_O_8_, the independent tetra­methyl 9,10-dihydro-9,10-dioxoanthracene-2,3,6,7-tetra­carboxyl­ate, (I), and tetra­methyl anthracene-2,3,6,7-tetra­carboxyl­ate, (II), components occupy separate crystallographic inversion centers. In (II), the dihedral angles between the mean aromatic plane and the two independent carboxyl­ate planes are 41.32 (10) and −38.35 (10)°. The methyl­carboxyl­ate groups of (I) are disordered, with each resolvable into two groups. In the least disordered carboxyl­ate, the apparent angles between the mean aromatic plane and the two partial carboxyl­ate planes [site occupations = 0.510 (3) and 0.490 (3)] are 16.8 (3) and 23.3 (3)°. In the highly disordered group, the apparent angles between the mean aromatic plane and the two partial carboxyl­ate planes [site occupations = 0.510 (3) and 0.490 (3)] are 78.3 (3) and −74.1 (3)°. In addition, this extreme disorder leads to an artificially elongated C(aromatic)—C(carbox­yl) bond.

## Related literature

For (I), see: Tarnchompoo *et al.* (1987 ▶). For (II), see: Luo & Hart (1988 ▶); Morris *et al.* (1994 ▶); Yanagimoto *et al.* (2006 ▶).
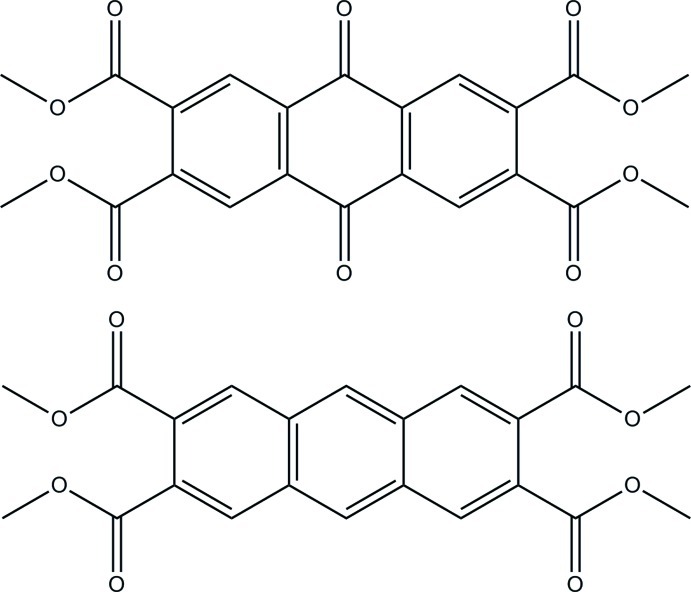


## Experimental

### 

#### Crystal data

C_22_H_16_O_10_·C_22_H_18_O_8_
*M* *_r_* = 850.71
Triclinic, 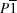

*a* = 8.2110 (3) Å
*b* = 9.5965 (3) Å
*c* = 12.0886 (5) Å
α = 86.281 (2)°
β = 81.514 (2)°
γ = 81.705 (2)°
*V* = 931.34 (6) Å^3^
*Z* = 1
Mo *K*α radiation
μ = 0.12 mm^−1^
*T* = 90 K
0.40 × 0.24 × 0.14 mm

#### Data collection

Nonius KappaCCD diffractometer
Absorption correction: multi-scan (*SCALEPACK*; Otwinowski & Minor, 1997 ▶) *T*_min_ = 0.954, *T*_max_ = 0.984
12654 measured reflections
7048 independent reflections
4579 reflections with *I* > 2σ(*I*)
*R*_int_ = 0.030

#### Refinement

*R*[*F* ^2^ > 2σ(*F* ^2^)] = 0.052
*wR*(*F* ^2^) = 0.154
*S* = 1.03
7048 reflections
326 parameters
H-atom parameters constrained
Δρ_max_ = 0.45 e Å^−3^
Δρ_min_ = −0.30 e Å^−3^

### 

Data collection: *COLLECT* (Nonius, 2000 ▶); cell refinement: *SCALEPACK* (Otwinowski & Minor, 1997 ▶); data reduction: *DENZO* (Otwinowski & Minor, 1997 ▶) and *SCALEPACK*; program(s) used to solve structure: *SHELXS97* (Sheldrick, 2008 ▶); program(s) used to refine structure: *SHELXL97* (Sheldrick, 2008 ▶); molecular graphics: *ORTEP-3 for Windows* (Farrugia, 1997 ▶); software used to prepare material for publication: *WinGX* (Farrugia, 1999 ▶).

## Supplementary Material

Crystal structure: contains datablock(s) global, I. DOI: 10.1107/S1600536812032424/pv2569sup1.cif

Structure factors: contains datablock(s) I. DOI: 10.1107/S1600536812032424/pv2569Isup2.hkl

Supplementary material file. DOI: 10.1107/S1600536812032424/pv2569Isup3.cml

Additional supplementary materials:  crystallographic information; 3D view; checkCIF report

## Figures and Tables

**Fig. 1.**
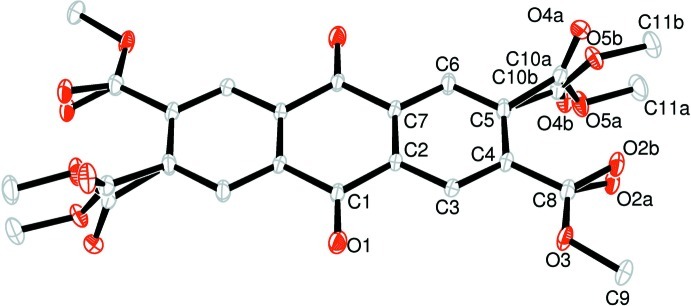
The molecular structure of (I) with the atom numbering scheme showing disorder. Displacement ellipsoids are drawn at the 30% probability level. H atoms were omitted for clarity.

**Fig. 2.**
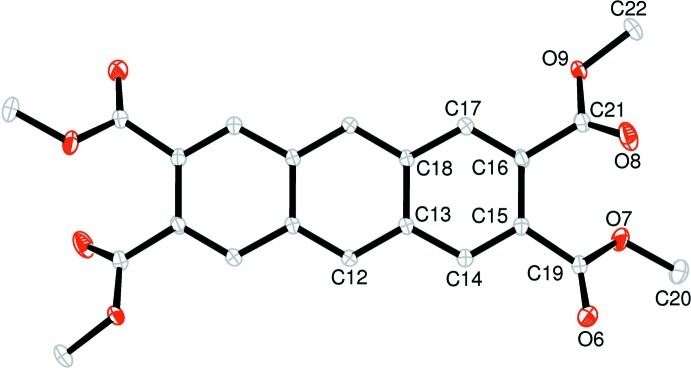
The molecular structure of (I) with the atom numbering scheme. Displacement ellipsoids are drawn at the 30% probability level. H atoms were omitted for clarity.

## supplementary crystallographic information

### Comment

The room temperature crystal structure of anthracene dedrivative (II) was determined by Morris *et al.* (1994). Along with pure crystals of (II), they obtained the co-crystalled product reported here. It is presumed that the anthraquinone derivative ((I)) was formed as an air oxidation product of (II).

The structure exhibits little aromatic ring stacking, with closest contacts C4···C18(at -*x*, -*y*, -*z*) 3.486 (2) and C2···C16(at -*x*, -*y*, -*z*) 3.575 (2) Å.

### Experimental

The synthesis of (II) was reported by Morris *et al.* (1994). It is presumed that the anthraquinone derivative ((I)) was formed as an air oxidation product of (II).

### Refinement

All H atoms were placed in calculated positions, guided by difference maps, with C—H bond distances 0.95 (aromatic-H) and 0.98 (methyl-H), displacement parameters *U*_iso_=1.2*U*_eq_ (aromatic C) and 1.5*U*_eq_ (methyl-C), and thereafter refined as riding.

The site occupation factor for disordered atoms O2A and O2B were constrained to be X1 and 1 - X1 respectively, and both atoms were constrained to have the same anisotropic displacement paramters; X1 was refined to a value of 0.510 (3). Likewise, site occupation factor X2 was independently refined to a value of 0.510 (3) for disordered methylcarboxylate O4A, C10A, O5A, C11A (1 - X2 for O4B, C10B, O5B, C11B).

### Crystal data

**Table:**

C_22_H_16_O_10_·C_22_H_18_O_8_	*Z* = 1
*M**_r_* = 850.71	*F*(000) = 442
Triclinic, *P*1	*D*_x_ = 1.517 Mg m^−^^3^
Hall symbol: -P 1	Mo *K*α radiation, λ = 0.71073 Å
*a* = 8.2110 (3) Å	Cell parameters from 6290 reflections
*b* = 9.5965 (3) Å	θ = 2.6–33.1°
*c* = 12.0886 (5) Å	µ = 0.12 mm^−^^1^
α = 86.281 (2)°	*T* = 90 K
β = 81.514 (2)°	Plate, yellow
γ = 81.705 (2)°	0.40 × 0.24 × 0.14 mm
*V* = 931.34 (6) Å^3^	

### Data collection

**Table:**

Nonius KappaCCD diffractometer	7048 independent reflections
Radiation source: sealed tube	4579 reflections with *I* > 2σ(*I*)
Horizonally mounted graphite crystal monochromator	*R*_int_ = 0.030
Detector resolution: 9 pixels mm^-1^	θ_max_ = 33.1°, θ_min_ = 2.7°
CCD rotation images, thick slices scans	*h* = −11→12
Absorption correction: multi-scan (*SCALEPACK*; Otwinowski & Minor, 1997)	*k* = −14→14
*T*_min_ = 0.954, *T*_max_ = 0.984	*l* = −18→18
12654 measured reflections	

### Refinement

**Table:**

Refinement on *F*^2^	Primary atom site location: structure-invariant direct methods
Least-squares matrix: full	Secondary atom site location: difference Fourier map
*R*[*F*^2^ > 2σ(*F*^2^)] = 0.052	Hydrogen site location: inferred from neighbouring sites
*wR*(*F*^2^) = 0.154	H-atom parameters constrained
*S* = 1.03	*w* = 1/[σ^2^(*F*_o_^2^) + (0.0865*P*)^2^] where *P* = (*F*_o_^2^ + 2*F*_c_^2^)/3
7048 reflections	(Δ/σ)_max_ = 0.001
326 parameters	Δρ_max_ = 0.45 e Å^−^^3^
0 restraints	Δρ_min_ = −0.30 e Å^−^^3^
3 constraints	

### Special details

**Table:**

Geometry. All e.s.d.'s (except the e.s.d. in the dihedral angle between two l.s. planes) are estimated using the full covariance matrix. The cell e.s.d.'s are taken into account individually in the estimation of e.s.d.'s in distances, angles and torsion angles; correlations between e.s.d.'s in cell parameters are only used when they are defined by crystal symmetry. An approximate (isotropic) treatment of cell e.s.d.'s is used for estimating e.s.d.'s involving l.s. planes.

### Fractional atomic coordinates and isotropic or equivalent isotropic displacement parameters (Å^2^)

**Table:**

	*x*	*y*	*z*	*U*_iso_*/*U*_eq_	Occ. (<1)
C1	0.17551 (13)	−0.04598 (11)	0.45522 (9)	0.0160 (2)	
C2	0.03630 (13)	−0.08672 (11)	0.40307 (9)	0.0146 (2)	
C3	0.07556 (13)	−0.17164 (11)	0.31043 (9)	0.0165 (2)	
H3	0.1885	−0.198	0.28	0.02*	
C4	−0.05059 (14)	−0.21760 (12)	0.26265 (10)	0.0185 (2)	
C5	−0.21709 (14)	−0.17679 (13)	0.30674 (11)	0.0231 (3)	
C6	−0.25640 (14)	−0.08911 (13)	0.39726 (11)	0.0228 (3)	
H6	−0.3694	−0.0592	0.4256	0.027*	
C7	−0.12956 (13)	−0.04518 (11)	0.44636 (9)	0.0160 (2)	
C8	−0.00953 (15)	−0.31246 (14)	0.16572 (11)	0.0256 (3)	
O2A	−0.1090 (3)	−0.3944 (3)	0.1466 (2)	0.0274 (4)	0.510 (3)
O2B	−0.1136 (3)	−0.3369 (3)	0.1098 (2)	0.0274 (4)	0.490 (3)
C9	0.20164 (16)	−0.42773 (13)	0.03751 (11)	0.0258 (3)	
H9A	0.1959	−0.5242	0.0682	0.039*	
H9B	0.316	−0.4188	0.0039	0.039*	
H9C	0.1277	−0.4063	−0.0198	0.039*	
C10A	−0.3594 (5)	−0.2057 (4)	0.2470 (3)	0.0179 (7)	0.490 (3)
O4A	−0.4377 (3)	−0.1268 (3)	0.18701 (17)	0.0208 (5)	0.490 (3)
O5A	−0.3849 (4)	−0.3372 (3)	0.2787 (3)	0.0264 (5)	0.490 (3)
C11A	−0.4895 (4)	−0.3982 (3)	0.2144 (3)	0.0336 (8)	0.490 (3)
H11A	−0.6012	−0.3958	0.2568	0.05*	0.490 (3)
H11B	−0.4424	−0.496	0.1996	0.05*	0.490 (3)
H11C	−0.496	−0.344	0.1433	0.05*	0.490 (3)
C10B	−0.3558 (5)	−0.2516 (4)	0.2748 (4)	0.0188 (7)	0.510 (3)
O4B	−0.4236 (3)	−0.3413 (3)	0.3294 (2)	0.0263 (5)	0.510 (3)
O5B	−0.3920 (3)	−0.1937 (3)	0.17661 (18)	0.0219 (5)	0.510 (3)
C11B	−0.4997 (3)	−0.2659 (3)	0.1233 (2)	0.0274 (6)	0.510 (3)
H11D	−0.5511	−0.3327	0.1774	0.041*	0.510 (3)
H11E	−0.4345	−0.317	0.0602	0.041*	0.510 (3)
H11F	−0.5866	−0.197	0.0959	0.041*	0.510 (3)
O1	0.31995 (10)	−0.08674 (9)	0.41989 (8)	0.0246 (2)	
O3	0.15036 (10)	−0.32984 (9)	0.12660 (7)	0.02195 (19)	
C12	−0.16785 (13)	0.04142 (11)	0.04459 (9)	0.0160 (2)	
H12	−0.2806	0.0698	0.0743	0.019*	
C13	−0.12949 (13)	−0.04723 (11)	−0.04583 (9)	0.0151 (2)	
C14	−0.25614 (13)	−0.09921 (12)	−0.09405 (10)	0.0178 (2)	
H14	−0.3693	−0.0725	−0.0644	0.021*	
C15	−0.21832 (13)	−0.18678 (12)	−0.18222 (9)	0.0163 (2)	
C16	−0.04783 (13)	−0.22772 (11)	−0.22822 (9)	0.0147 (2)	
C17	0.07649 (13)	−0.17920 (11)	−0.18403 (9)	0.0155 (2)	
H17	0.1889	−0.2062	−0.2154	0.019*	
C18	0.04030 (13)	−0.08847 (11)	−0.09143 (9)	0.0145 (2)	
C19	−0.36209 (14)	−0.23079 (12)	−0.22755 (10)	0.0192 (2)	
C20	−0.46657 (16)	−0.28536 (17)	−0.38791 (12)	0.0338 (3)	
H20A	−0.4857	−0.3776	−0.3528	0.051*	
H20B	−0.4317	−0.2946	−0.4685	0.051*	
H20C	−0.5695	−0.2192	−0.3756	0.051*	
C21	−0.00127 (13)	−0.33641 (12)	−0.31496 (9)	0.0167 (2)	
C22	0.19237 (15)	−0.41180 (12)	−0.47134 (10)	0.0221 (2)	
H22A	0.2222	−0.5027	−0.4325	0.033*	
H22B	0.2911	−0.3838	−0.5185	0.033*	
H22C	0.1069	−0.4203	−0.5183	0.033*	
O6	−0.48764 (10)	−0.25750 (10)	−0.16986 (8)	0.0285 (2)	
O7	−0.33807 (10)	−0.23321 (10)	−0.33907 (7)	0.0266 (2)	
O8	−0.06719 (12)	−0.44073 (9)	−0.31555 (8)	0.0276 (2)	
O9	0.12862 (9)	−0.30601 (8)	−0.38957 (7)	0.01791 (17)	

### Atomic displacement parameters (Å^2^)

**Table:**

	*U*^11^	*U*^22^	*U*^33^	*U*^12^	*U*^13^	*U*^23^
C1	0.0173 (5)	0.0168 (5)	0.0150 (5)	−0.0030 (4)	−0.0027 (4)	−0.0062 (4)
C2	0.0171 (5)	0.0146 (5)	0.0131 (5)	−0.0021 (4)	−0.0038 (4)	−0.0047 (4)
C3	0.0179 (5)	0.0170 (5)	0.0150 (5)	0.0007 (4)	−0.0041 (4)	−0.0058 (4)
C4	0.0210 (5)	0.0191 (5)	0.0164 (5)	−0.0013 (4)	−0.0041 (4)	−0.0086 (4)
C5	0.0193 (5)	0.0290 (6)	0.0238 (6)	−0.0032 (4)	−0.0061 (4)	−0.0145 (5)
C6	0.0173 (5)	0.0296 (6)	0.0235 (6)	−0.0025 (4)	−0.0039 (4)	−0.0153 (5)
C7	0.0178 (5)	0.0155 (5)	0.0157 (5)	−0.0017 (4)	−0.0032 (4)	−0.0069 (4)
C8	0.0228 (6)	0.0311 (6)	0.0244 (6)	0.0008 (5)	−0.0061 (5)	−0.0164 (5)
O2A	0.0273 (6)	0.0296 (11)	0.0287 (12)	−0.0019 (9)	−0.0094 (8)	−0.0177 (7)
O2B	0.0273 (6)	0.0296 (11)	0.0287 (12)	−0.0019 (9)	−0.0094 (8)	−0.0177 (7)
C9	0.0321 (6)	0.0267 (6)	0.0190 (6)	−0.0009 (5)	−0.0019 (5)	−0.0130 (5)
C10A	0.0212 (13)	0.0160 (17)	0.018 (2)	−0.0030 (14)	−0.0031 (13)	−0.0074 (12)
O4A	0.0196 (9)	0.0201 (11)	0.0237 (10)	−0.0005 (8)	−0.0080 (7)	−0.0024 (8)
O5A	0.0400 (15)	0.0175 (10)	0.0285 (14)	−0.0119 (9)	−0.0197 (12)	0.0009 (10)
C11A	0.0434 (17)	0.0230 (13)	0.0428 (18)	−0.0121 (11)	−0.0256 (14)	−0.0032 (12)
C10B	0.0191 (12)	0.022 (2)	0.0170 (18)	−0.0014 (15)	−0.0046 (12)	−0.0077 (13)
O4B	0.0326 (11)	0.0298 (11)	0.0209 (12)	−0.0155 (8)	−0.0054 (10)	−0.0043 (10)
O5B	0.0216 (10)	0.0268 (12)	0.0208 (10)	−0.0071 (9)	−0.0112 (7)	−0.0003 (8)
C11B	0.0249 (12)	0.0382 (14)	0.0236 (13)	−0.0110 (10)	−0.0096 (10)	−0.0052 (10)
O1	0.0178 (4)	0.0311 (5)	0.0257 (5)	−0.0031 (3)	−0.0004 (3)	−0.0152 (4)
O3	0.0260 (4)	0.0240 (4)	0.0168 (4)	−0.0049 (3)	0.0000 (3)	−0.0115 (3)
C12	0.0122 (5)	0.0204 (5)	0.0159 (5)	−0.0019 (4)	−0.0008 (4)	−0.0067 (4)
C13	0.0145 (5)	0.0173 (5)	0.0140 (5)	−0.0025 (4)	−0.0014 (4)	−0.0053 (4)
C14	0.0144 (5)	0.0226 (5)	0.0174 (5)	−0.0038 (4)	−0.0014 (4)	−0.0076 (4)
C15	0.0156 (5)	0.0192 (5)	0.0153 (5)	−0.0047 (4)	−0.0016 (4)	−0.0051 (4)
C16	0.0172 (5)	0.0152 (5)	0.0120 (5)	−0.0029 (4)	−0.0009 (4)	−0.0039 (4)
C17	0.0146 (5)	0.0178 (5)	0.0140 (5)	−0.0019 (4)	−0.0008 (4)	−0.0046 (4)
C18	0.0146 (5)	0.0167 (5)	0.0129 (5)	−0.0028 (4)	−0.0016 (4)	−0.0046 (4)
C19	0.0170 (5)	0.0224 (5)	0.0196 (6)	−0.0032 (4)	−0.0029 (4)	−0.0098 (4)
C20	0.0235 (6)	0.0548 (9)	0.0280 (7)	−0.0077 (6)	−0.0098 (5)	−0.0189 (6)
C21	0.0187 (5)	0.0183 (5)	0.0137 (5)	−0.0024 (4)	−0.0030 (4)	−0.0041 (4)
C22	0.0260 (6)	0.0225 (5)	0.0171 (6)	−0.0007 (4)	0.0006 (4)	−0.0104 (4)
O6	0.0196 (4)	0.0419 (5)	0.0265 (5)	−0.0119 (4)	0.0014 (3)	−0.0144 (4)
O7	0.0222 (4)	0.0437 (5)	0.0180 (4)	−0.0107 (4)	−0.0054 (3)	−0.0107 (4)
O8	0.0393 (5)	0.0219 (4)	0.0228 (5)	−0.0140 (4)	0.0054 (4)	−0.0093 (3)
O9	0.0181 (4)	0.0202 (4)	0.0160 (4)	−0.0039 (3)	0.0007 (3)	−0.0089 (3)

### Geometric parameters (Å, º)

**Table:**

C1—O1	1.2166 (13)	O5B—C11B	1.442 (3)
C1—C2	1.4934 (14)	C11B—H11D	0.98
C1—C7^i^	1.4935 (14)	C11B—H11E	0.98
C2—C7	1.3956 (15)	C11B—H11F	0.98
C2—C3	1.3994 (14)	C12—C13	1.3998 (14)
C3—C4	1.3938 (15)	C12—C18^ii^	1.4021 (14)
C3—H3	0.95	C12—H12	0.95
C4—C5	1.4015 (16)	C13—C14	1.4270 (14)
C4—C8	1.4995 (15)	C13—C18	1.4305 (15)
C5—C6	1.3951 (16)	C14—C15	1.3718 (15)
C5—C10A	1.527 (4)	C14—H14	0.95
C5—C10B	1.537 (4)	C15—C16	1.4364 (15)
C6—C7	1.3987 (15)	C15—C19	1.4957 (15)
C6—H6	0.95	C16—C17	1.3691 (14)
C7—C1^i^	1.4935 (14)	C16—C21	1.4974 (14)
C8—O2B	1.221 (3)	C17—C18	1.4329 (14)
C8—O2A	1.267 (3)	C17—H17	0.95
C8—O3	1.3187 (14)	C18—C12^ii^	1.4021 (14)
C9—O3	1.4501 (13)	C19—O6	1.2064 (14)
C9—H9A	0.98	C19—O7	1.3346 (15)
C9—H9B	0.98	C20—O7	1.4446 (13)
C9—H9C	0.98	C20—H20A	0.98
C10A—O4A	1.199 (4)	C20—H20B	0.98
C10A—O5A	1.331 (4)	C20—H20C	0.98
O5A—C11A	1.444 (3)	C21—O8	1.2056 (13)
C11A—H11A	0.98	C21—O9	1.3445 (13)
C11A—H11B	0.98	C22—O9	1.4512 (13)
C11A—H11C	0.98	C22—H22A	0.98
C10B—O4B	1.201 (4)	C22—H22B	0.98
C10B—O5B	1.333 (4)	C22—H22C	0.98
			
O1—C1—C2	121.43 (9)	C8—O3—C9	115.83 (9)
O1—C1—C7^i^	121.49 (9)	C13—C12—C18^ii^	120.19 (10)
C2—C1—C7^i^	117.08 (9)	C13—C12—H12	119.9
C7—C2—C3	120.05 (9)	C18^ii^—C12—H12	119.9
C7—C2—C1	121.57 (9)	C12—C13—C14	121.62 (9)
C3—C2—C1	118.37 (9)	C12—C13—C18	119.87 (9)
C4—C3—C2	120.16 (10)	C14—C13—C18	118.51 (9)
C4—C3—H3	119.9	C15—C14—C13	121.55 (10)
C2—C3—H3	119.9	C15—C14—H14	119.2
C3—C4—C5	119.74 (10)	C13—C14—H14	119.2
C3—C4—C8	120.53 (10)	C14—C15—C16	119.90 (9)
C5—C4—C8	119.72 (10)	C14—C15—C19	116.52 (10)
C6—C5—C4	120.10 (10)	C16—C15—C19	123.55 (9)
C6—C5—C10A	117.66 (17)	C17—C16—C15	119.93 (9)
C4—C5—C10A	121.49 (17)	C17—C16—C21	118.53 (9)
C6—C5—C10B	117.84 (17)	C15—C16—C21	121.13 (9)
C4—C5—C10B	120.72 (16)	C16—C17—C18	121.24 (10)
C5—C6—C7	120.05 (10)	C16—C17—H17	119.4
C5—C6—H6	120	C18—C17—H17	119.4
C7—C6—H6	120	C12^ii^—C18—C13	119.93 (9)
C2—C7—C6	119.86 (10)	C12^ii^—C18—C17	121.20 (10)
C2—C7—C1^i^	121.33 (9)	C13—C18—C17	118.86 (9)
C6—C7—C1^i^	118.81 (9)	O6—C19—O7	123.75 (10)
O2B—C8—O3	121.61 (16)	O6—C19—C15	123.71 (11)
O2A—C8—O3	123.52 (14)	O7—C19—C15	112.51 (10)
O2B—C8—C4	122.73 (15)	O7—C20—H20A	109.5
O2A—C8—C4	121.19 (15)	O7—C20—H20B	109.5
O3—C8—C4	112.55 (9)	H20A—C20—H20B	109.5
O3—C9—H9A	109.5	O7—C20—H20C	109.5
O3—C9—H9B	109.5	H20A—C20—H20C	109.5
H9A—C9—H9B	109.5	H20B—C20—H20C	109.5
O3—C9—H9C	109.5	O8—C21—O9	124.17 (10)
H9A—C9—H9C	109.5	O8—C21—C16	124.51 (10)
H9B—C9—H9C	109.5	O9—C21—C16	111.26 (9)
O4A—C10A—O5A	126.1 (4)	O9—C22—H22A	109.5
O4A—C10A—C5	128.3 (3)	O9—C22—H22B	109.5
O5A—C10A—C5	105.6 (3)	H22A—C22—H22B	109.5
C10A—O5A—C11A	115.2 (3)	O9—C22—H22C	109.5
O4B—C10B—O5B	126.2 (3)	H22A—C22—H22C	109.5
O4B—C10B—C5	126.9 (3)	H22B—C22—H22C	109.5
O5B—C10B—C5	106.9 (3)	C19—O7—C20	115.38 (10)
C10B—O5B—C11B	115.1 (2)	C21—O9—C22	115.34 (8)
			
O1—C1—C2—C7	177.20 (11)	C10A—C5—C10B—O4B	−161.3 (11)
C7^i^—C1—C2—C7	−1.92 (18)	C6—C5—C10B—O5B	110.9 (3)
O1—C1—C2—C3	−1.13 (17)	C4—C5—C10B—O5B	−82.3 (3)
C7^i^—C1—C2—C3	179.75 (10)	C10A—C5—C10B—O5B	16.0 (6)
C7—C2—C3—C4	−1.43 (17)	O4B—C10B—O5B—C11B	−12.7 (5)
C1—C2—C3—C4	176.91 (10)	C5—C10B—O5B—C11B	169.9 (2)
C2—C3—C4—C5	0.90 (18)	O2B—C8—O3—C9	−23.4 (2)
C2—C3—C4—C8	−178.00 (11)	O2A—C8—O3—C9	14.7 (2)
C3—C4—C5—C6	0.8 (2)	C4—C8—O3—C9	176.06 (10)
C8—C4—C5—C6	179.69 (12)	C18^ii^—C12—C13—C14	178.77 (11)
C3—C4—C5—C10A	170.7 (2)	C18^ii^—C12—C13—C18	−0.79 (18)
C8—C4—C5—C10A	−10.4 (3)	C12—C13—C14—C15	−179.59 (11)
C3—C4—C5—C10B	−165.8 (2)	C18—C13—C14—C15	−0.04 (17)
C8—C4—C5—C10B	13.2 (3)	C13—C14—C15—C16	−0.19 (18)
C4—C5—C6—C7	−1.9 (2)	C13—C14—C15—C19	−178.28 (10)
C10A—C5—C6—C7	−172.2 (2)	C14—C15—C16—C17	−0.04 (17)
C10B—C5—C6—C7	165.0 (2)	C19—C15—C16—C17	177.91 (11)
C3—C2—C7—C6	0.28 (17)	C14—C15—C16—C21	172.59 (11)
C1—C2—C7—C6	−178.01 (11)	C19—C15—C16—C21	−9.46 (17)
C3—C2—C7—C1^i^	−179.70 (10)	C15—C16—C17—C18	0.50 (17)
C1—C2—C7—C1^i^	2.00 (18)	C21—C16—C17—C18	−172.32 (10)
C5—C6—C7—C2	1.40 (19)	C12—C13—C18—C12^ii^	0.79 (18)
C5—C6—C7—C1^i^	−178.62 (12)	C14—C13—C18—C12^ii^	−178.78 (10)
C3—C4—C8—O2B	−167.8 (2)	C12—C13—C18—C17	−179.96 (10)
C5—C4—C8—O2B	13.3 (3)	C14—C13—C18—C17	0.48 (16)
C3—C4—C8—O2A	154.37 (19)	C16—C17—C18—C12^ii^	178.53 (11)
C5—C4—C8—O2A	−24.5 (2)	C16—C17—C18—C13	−0.72 (17)
C3—C4—C8—O3	−7.51 (18)	C14—C15—C19—O6	−37.99 (17)
C5—C4—C8—O3	173.58 (12)	C16—C15—C19—O6	143.99 (13)
C6—C5—C10A—O4A	71.8 (4)	C14—C15—C19—O7	140.16 (11)
C4—C5—C10A—O4A	−98.4 (4)	C16—C15—C19—O7	−37.85 (16)
C10B—C5—C10A—O4A	167.6 (11)	C17—C16—C21—O8	134.54 (13)
C6—C5—C10A—O5A	−105.8 (3)	C15—C16—C21—O8	−38.19 (17)
C4—C5—C10A—O5A	84.1 (3)	C17—C16—C21—O9	−42.72 (14)
C10B—C5—C10A—O5A	−9.9 (6)	C15—C16—C21—O9	144.55 (11)
O4A—C10A—O5A—C11A	15.1 (6)	O6—C19—O7—C20	−6.45 (18)
C5—C10A—O5A—C11A	−167.3 (3)	C15—C19—O7—C20	175.39 (10)
C6—C5—C10B—O4B	−66.5 (4)	O8—C21—O9—C22	−2.47 (17)
C4—C5—C10B—O4B	100.4 (4)	C16—C21—O9—C22	174.80 (9)

Symmetry codes: (i) −*x*, −*y*, −*z*+1; (ii) −*x*, −*y*, −*z*.
